# DEPENDENT INDEPENDENCE: REFRAMING AGING AND CAREGIVING AFTER CHINA’S ONE-CHILD POLICY

**DOI:** 10.1093/geroni/igz038.3104

**Published:** 2019-11-08

**Authors:** Chen Chi

**Affiliations:** University of Chicago, Chicago, Illinois, United States

## Abstract

Global aging has led to substantive demands for caregiving. In China, the country’s birth control policy and recent economic downturn have exacerbated the situation. Given the decreasing care they have received, older Chinese online users 65-74 years of age from single-child families, however, have demonstrated more positive attitudes than negative ones—expressing satisfaction and speaking highly of their adult children’s filial care. Why older Chinese come to appreciate their adult children’s filial performance? Building upon the concept of “regeneration” (Cole and Durham, 2007), I propose the term “dependent independence” to highlight the mutually constitutive parent-adult children relations in China’s single-child families. Employing a relational approach to aging and care in China, I analyze online posts from China’s most populous information-sharing platform, Zhihu. As the major cause of the care crisis in China, the One-Child Policy, I argue, creates the solution at the same time by modernizing Chinese families such that new care values and preference, specifically affective bonds and independence, have become dominant. I first demonstrate that this sample of older Chinese adults have shifted care preferences from physical support to affective bonds. I then analyze their reformulation of care values from servility to older adults to adult children’s independence and individual success. Revealing the changing values and preference of caregiving and family relations in China, this paper reminds us of what kind of future we aspire to, and what kind of values we cling to no matter as an adult child or an older adult.

